# Trans-Mesenteric Hernia in Infants: Report of Two Cases

**Published:** 2014-07-10

**Authors:** Aziz Elmadi, M Lechqar, I El Biache, S Tenkorang, K Khattala, M Rami, Y Bouabdallah

**Affiliations:** Department of Pediatric Surgery, University Hospital Hassan II Fez, MORROCO

**Keywords:** Internal hernia, Trans-mesenteric, Neonate, Obstruction, Bowel

## Abstract

Internal hernias are rare causes of intestinal obstruction in children. Trans-mesenteric hernia remains the most common form. We report transmesenteric hernia in a neonate and infant presented with intestinal obstruction.

## INTRODUCTION

An internal hernia is defined as the protrusion of viscera through peritoneal mesenteric aperture. Transmesenteric hernia is a form of internal hernia through a congenital defect in the mesentery. It is a rare but serious cause of intestinal obstruction in children. It represents only 5 to 10% of internal hernias [1]. Its complications have often been described in children. Symptoms are non-specific. Plain abdomen, and abdominal CT scan are useful for diagnosis of a transmesenteric hernia, but an accurate preoperative diagnosis is rarely made [1-3]. We herein report two cases of transmesenteric hernia. 

## CASE SERIES

**Case 1:** A 41-day-old infant presented with abdominal distension, bilious vomiting and fever occurring two days before admission. Clinical examination found a hypotonic infant, pale with fever at 40°C, abdominal distension, and blood stained finger on rectal examination. The remainder of the physical examination was unremarkable. Diagnosis of intestinal obstruction was raised. A biological assessment was performed revealing leukocytosis and elevated C-reactive protein. Abdominal radiography showed multiple air fluid levels. Abdominal ultrasound gave suspicion of volvulus of small bowel. After resuscitation measures, surgical exploration divulged hernia of the mesentery of the terminal ileum with intestinal volvulus that was still viable. We therefore proceeded with reduction of the bowel, small bowel untwisting, and closure of the mesenteric defect (Fig. 1). Postoperative follow-up was uneventful. The patient is on follow-up for 18 months.

**Figure:**
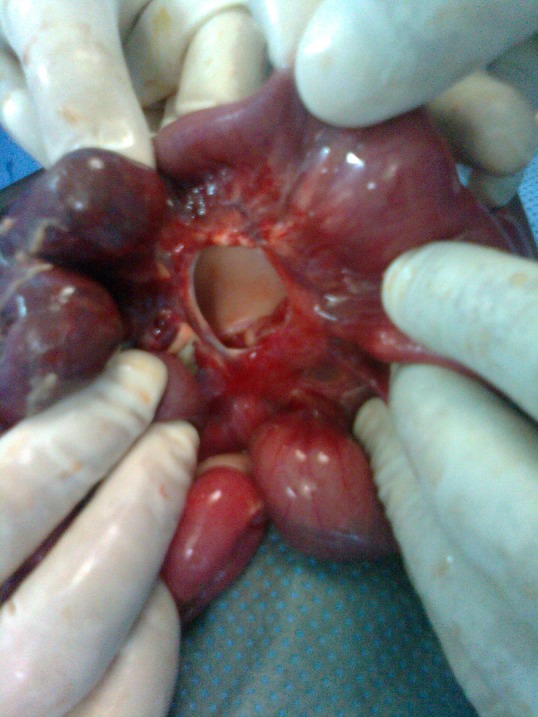
Figure 1: Defect in the mesentery of the jejunum measuring 2cm in diameter.

**Case 2:**A 4-day-old newborn presented with bilious vomiting and abdominal distention. Abdominal radiograph showed multiple air-fluid levels giving an impression of small bowel obstruction. All laboratory tests were within normal limits. Surgical exploration found a trans-mesenteric hernia and gangrenous small bowel (Fig. 2) located 38 cm from the ligament of Treitz. Resection of the necrotic loop followed by primary anastomosis was done. The mesenteric defect was repaired. Postoperative recovery and follow-up were uneventful.

**Figure:**
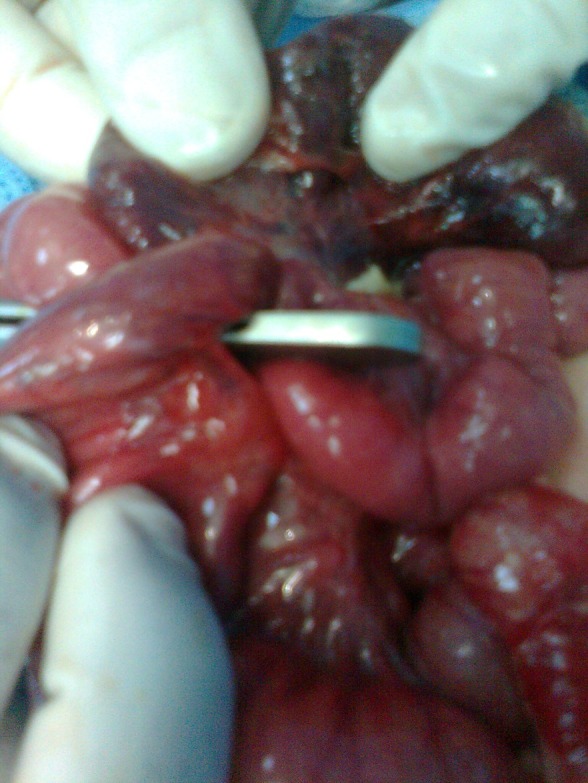
Figure 2: Presence of intestinal necrosis and mesenteric defect.

## DISCUSSION

Transmesenteric hernias are a rare cause of acute small bowel obstruction in children; about 35% of cases occur during childhood [2]. They are often located near the ileocecal junction [1,2]. The etiology is attributed to a prenatal intestinal ischemia leading to thinning of the mesenteric walls associated with mesenteric small bowel atresia in 5.5 ℅ of the pediatric population [3]. In neonates, most patients remain relatively asymptomatic until they are started on enteral feeds when patients generally present with persistent vomiting and abdominal distention; few patients present in neonatal life with intestinal obstruction as found in our case [1].

Radiological assessment by abdominal radiograph in children shows air-fluid levels relative to the small bowel obstruction. CT scan shows signs of small-bowel obstruction; clustering of small bowel; stretched, displaced, crowded, and engorged mesenteric vessels; and displacement of other bowel segments [4]. Surgical treatment has no particular difficulty. Treatment is based on the reduction of the hernial contents, resection of necrotic bowel, primary anastomosis or, rarely enterostomy, and correction of the anatomic defect [5]. Prognosis of trans-mesenteric hernia is usually good if diagnosis is considered earlier and treatment is performed rapidly. The mortality rate associated with incarceration of the bowel is about 15%, but in the presence of gangrene of the bowel, the mortality rate is more than 50% [6]. In our second case, there was bowel gangrene owing to associated volvulus of small bowel which needed resection and primary anastomosis.

## Conclusion

Trans-mesenteric hernia is a rare cause of intestinal obstruction in neonates and infants. Preoperative diagnosis is extremely difficult. Treatment involves repositioning of the intestinal loops at all times and prudent closure of the peritoneal orifice in question.

## Footnotes

**Source of Support:** Nil

**Conflict of Interest:** None

